# Annotations

**Published:** 1901-01-05

**Authors:** 


### ANNOTATIONS.

### The Census and Density of Population.

In these days of large cities it is well to be reminded how very recent is their growth; for in truth there has not yet been time to ascertain with anything approaching accuracy what is the necessary and essential influence of town life either upon the health and longevity of the individual or upon the racial stamina of the community, although so far as we know anything the influence appears to be bad. In a paper read before the Royal Statistical Society Mr. T. A. Welton points out, among other things, that in the year 1801 no town outside London had a population reaching to 100,000, while London itself contained only 922,000 inhabitants. Thus we see that, complicating the apparently simple comparison which might be drawn between the vital statistics of town and country in any one year, is the fact that we are in the midst of a constant process of change in regard to the distribution of the population, and that the higher mortality of towns may be due not wholly to any essential attributes of town life, but partly to the fact of the people being thrown among conditions to which they have not yet become hereditarily acclimatised, and partly again—largely, as we think—to the fact that owing to the steady inpouring of the people the towns have all along been too small for their population. This is but another way of saying that the higher mortality which so far has accompanied the aggregation of the people into towns is to a large extent due to one factor in town life—namely, overcrowding in the dwelling-houses—and it is to be hoped that when the next census is taken measures will be adopted to show the extent to which such overcrowding exists.

### Sanitation and the Motorists.

If, as we believe to be the case, the motor car has a great future before it, the whole question of the pavement of our streets will have to be reconsidered. Regard for the horse has hitherto dominated the whole question of roadways. Obviously the smoother and the harder the surface of a road the less will be the power required to move wheeled vehicles upon it. But unfortunately the harder and the smoother the surface of the road the more difficult it is for the horse to obtain a foothold. Hence the objections so often raised to the use of asphalte, notwithstanding that it is in itself an almost ideal material from which to construct road surfaces. It is hard, it is smooth, it is durable, it is capable of being repaired bit by bit without tearing up the whole roadway, and above all it can be kept clean. But the horses slip, and this has been a fatal obstacle. With the introduction of the motor, however, the conditions may be altered, and if we consider the immense advantages which asphalte possesses from a sanitary point of view and the economy in motive power which must result from its smoothness, we may be quite sure that in proportion as the horse becomes non-essential the use of asphalte or some similar smooth and cleanly road surface will come more and more into vogue in all towns where the gradients allow of its employment. It is the sanitary aspect of asphalte pavement which especially appeals to us. So far it seems to be the only form of road surface which is capable of being kept decently clean, and this is a matter of the first importance. It is not a mere question of taste and æsthetics. If we are to accept the conclusions arrived at by Dr. Waldo, medical officer of health to the parish of St. George the Martyr—conclusions arrived at after very careful investigation of the facts—a very considerable proportion of the mortality from diarrhœa, one of the most fatal of diseases among town children, is due to the pollution of milk and other articles of food with street dust, in other words with dried horse dung highly charged with deleterious micro-organisms. Filthy street dust we must always have with us, unless we have streets that we can wash. It is probably to the facility with which asphalte can be washed that we must look for the explanation of the largely diminished death-rates which have been noted in certain towns after the introduction of this material as a paving. It is claimed that in New York the death-rate has gone down from 38·37 per 1,000 in 1892, to 26 per 1,000 in 1896, when the cleaner streets due to the large extension of asphalte pavement had begun to show their influence upon the health of the population; and although we can hardly doubt that the introduction of asphalte was, in this case, only one item in a general improvement in sanitary conditions, the benefits which have already resulted in London, in Paris, and in many other large towns, from the use of asphalte in courts and alleys has been such as to show how great would be the advantage of its more extended use. Not until all our streets are provided with a hard smooth surface which can be thoroughly cleansed shall we even begin to see what town life might be made. It is to the motor car that we must look to bring this much-needed reform within the range of practical politics.

### "Open Sesame."

The Sesame Club, which was founded four years ago with the purpose of turning the attention of girls to preparation for home-life, has decided to open a "Sesame House" in London. We have colleges for the training of girls in all professions except the one which the vast majority of them ultimately enter, domestic life. By domestic life we do not mean that of servants only. Ignorant as servants often are of their work, they are not a bit more ignorant than their mistresses, and an incompetent mistress is a far more contemptible personage than an untrained servant. What would be the result, in any other business, of the employer giving instructions which could not be carried out, ordering goods which were unattainable at that season of the year, demanding results for which no machinery was provided? Such conduct leads to bankruptcy in the business world, and if it does not result in exactly the same in the home, it leads to a lack of comfort in return for expense, in waste, irritation, and mutual contempt instead of mutual respect between mistress and servant. It says much for our girls that after a year or two's bungling, they so often become competent housekeepers; it says more for them, that entering married life with as little knowledge as they generally possess, they make good mothers. But, unless they are fortunate enough to have mothers to refer to, how often do they wish that they had had a little more knowledge of children's needs and nature before they took upon themselves the responsibility of motherhood! This training the Sesame House proposes to give. The course will include what is ordinarily known as housekeeping—that is the details of cooking and house-work, and the students will be instructed in ventilation, drainage, and sanitation, while such matters as the mysteries of plumbing and hot-water systems will be made plain to them. Such a training will harm no woman, whatever work she may finally engage in, and will be of the greatest practical use to the majority.

